# Effects of pyrroloquinoline quinone on noise-induced and age-related hearing loss in mice

**DOI:** 10.1038/s41598-022-19842-w

**Published:** 2022-09-23

**Authors:** Ying Gao, Teru Kamogashira, Chisato Fujimoto, Shinichi Iwasaki, Tatsuya Yamasoba

**Affiliations:** 1 Department of Otolaryngology and Head and Neck Surgery, Faculty of Medicine, University of Tokyo, 7-3-1, Hongo, Bunkyo-ku, Tokyo, 113-8655 Japan; 2 Department of Otolaryngology and Head and Neck Surgery, The Second Affiliated Hospital of Xi’an Jiaotong University, Xi’an, China; 3 Department of Otolaryngology and Head and Neck Surgery, Nagoya City University Graduate School of Medicine, Nagoya, Japan

**Keywords:** Cell biology, Neurological disorders, Preventive medicine

## Abstract

We investigated whether the oxidoreductase cofactor pyrroloquinoline quinone (PQQ) prevents noise-induced and age-related hearing loss (NIHL and ARHL) in mice. To assess NIHL, 8 week-old mice with and without PQQ administration were exposed to noise for 4 h. PQQ was orally administered for one week before and after noise exposure and subcutaneously once before noise exposure. For ARHL evaluation, mice were given drinking water with or without PQQ starting at 2 months of age. In the NIHL model, PQQ-treated mice had auditory brainstem response (ABR) thresholds of significantly reduced elevation at 8 kHz, a significantly increased number of hair cells at the basal turn, and significantly better maintained synapses beneath the inner hair cells compared to controls. In the ARHL model, PQQ significantly attenuated the age-related increase in ABR thresholds at 8 and 32 kHz at 10 months of age compared to controls. In addition, the hair cells, spiral ganglion cells, ribbon synapses, stria vascularis and nerve fibers were all significantly better maintained in PQQ-treated animals compared to controls at 10 months of age. These physiological and histological results demonstrate that PQQ protects the auditory system from NIHL and ARHL in mice.

## Introduction

Sensorineural hearing loss is one of the most common sensory disorders in humans and an important health issue for the elderly. Approximately 40% of the population over the age of 65 have hearing loss that interferes with their communication^1–3^. In humans, most cells in the cochlea including the hair cells (HCs) and spiral ganglion cells (SGCs) do not recover or regenerate once they are damaged or lost, and there is currently no effective treatment to recover hearing loss. Although hearing aids and cochlear implants are useful for those with permanent hearing loss^4^, the discovery of compounds that can protect the cochlea from damage or enhance the recovery is awaited.

Noise-induced hearing loss (NIHL) and age-related hearing loss (ARHL) are two of the most common causes of hearing loss in humans. NIHL occurs after loud noise exposure and is a problem for people working in noisy environments and for those who constantly listen to loud sounds^5^. The mechanisms of NIHL are mechanical, neural and metabolic^6–8^. NIHL is primarily caused by damage to the cochlear HCs and associated synaptopathy. Contributions to a temporary threshold shift (TTS) include reversible damage to the HC stereocilia or synapses beneath it, while a moderate TTS reflects protective purinergic hearing adaptation, which is thought to enable the highly sensitive hearing organ to maintain function even in loud sound, protecting the ear from acoustic overstimulation^9,10^. Permanent threshold shifts (PTS) represent permanent damage or loss of the HCs and synapses. The mechanisms of noise-induced HC damage include the generation and accumulation of reactive oxygen species (ROS) and the active stimulation of intracellular stress pathways, leading to programmed and/or necrotic cell death^9^. Permanent damage of the synapses beneath the HCs, cochlear nerve fibers and SGCs can also contribute to NIHL^9^. Other than non-pharmaceutical interventions such as earplugs and ear protectors, various agents have been evaluated to protect against or treat NIHL in animal models, including antioxidants, blood flow promoting drugs, neurotrophic factors and steroids^8,11^.

ARHL is characterized by an age-dependent decrease of auditory function, which is caused by the loss and dysfunction of the HCs, synapses, SGCs and stria vascularis (SV) in the cochlea^12^. ARHL is also associated with cognitive decline in community-dwelling older adults^13^. We previously proposed a putative mechanism of ARHL, in which the cumulative effect of oxidative stress could induce damage to macromolecules such as mitochondrial DNA (mtDNA) resulting in the accumulation of mtDNA mutations/deletions and the decline of mitochondrial function which both play important roles in inducing apoptosis of the cochlear cells^14^. Several ingredients have been reported to protect or ameliorate ARHL, including α-lipoic acid, coenzyme Q10 (CoQ10) and N-acetyl cysteine (NAC)^15^, which can scavenge ROS, mainly within mitochondria.

Pyrroloquinoline quinone (PQQ) is a redox cofactor of a number of prokaryotic dehydrogenases, and is present in plant and animal cells, as well as a variety of foods and drinks, including fermented soybeans (Japanese natto), tea, green peppers, parsley, kiwi fruit, and human milk^16,17^. PQQ is also known as a powerful antioxidant^18^. The effects of PQQ have been widely investigated, including its growth-promoting activity, anti-diabetic effect, anti-oxidative action and neuroprotective function^18^. PQQ is crucial for the degradation of the amino acid lysine in mice and acts as a mammalian redox cofactor in this reaction^19^. Dietary PQQ deprivation results in abnormal development, immune dysfunction, and decreased reproductive performance in animal models^18^. PQQ affects a wide range of genes, most notably those involved in mitochondrial-related functions^20,21^. Supplementary PQQ in the diet of mice and rats has been reported to improve mitochondrial content and lipid metabolism^22^ and the respiratory quotient by increasing the mitochondrial amount and function^23^. PQQ has been shown to prevent rotenone-induced neurotoxicity in Parkinson’s disease models by promoting mitochondrial function and regulating mitochondrial fission and fusion^24^. In humans, dietary supplementation with PQQ has been reported to recover the antioxidant potential, attenuate an inflammatory response, and increase urinary metabolites related to mitochondrial functions^21^.

Considering all the above findings, the mitochondrial and neural protective function of PQQ may also work in cochlear tissues, protecting them from NIHL and ARHL. In the current study, therefore, we investigated whether PQQ prevents NIHL and ARHL in mice.

## Results

### PQQ protected the hair cells and synapses from noise-induced hearing loss

**Figure 1:**
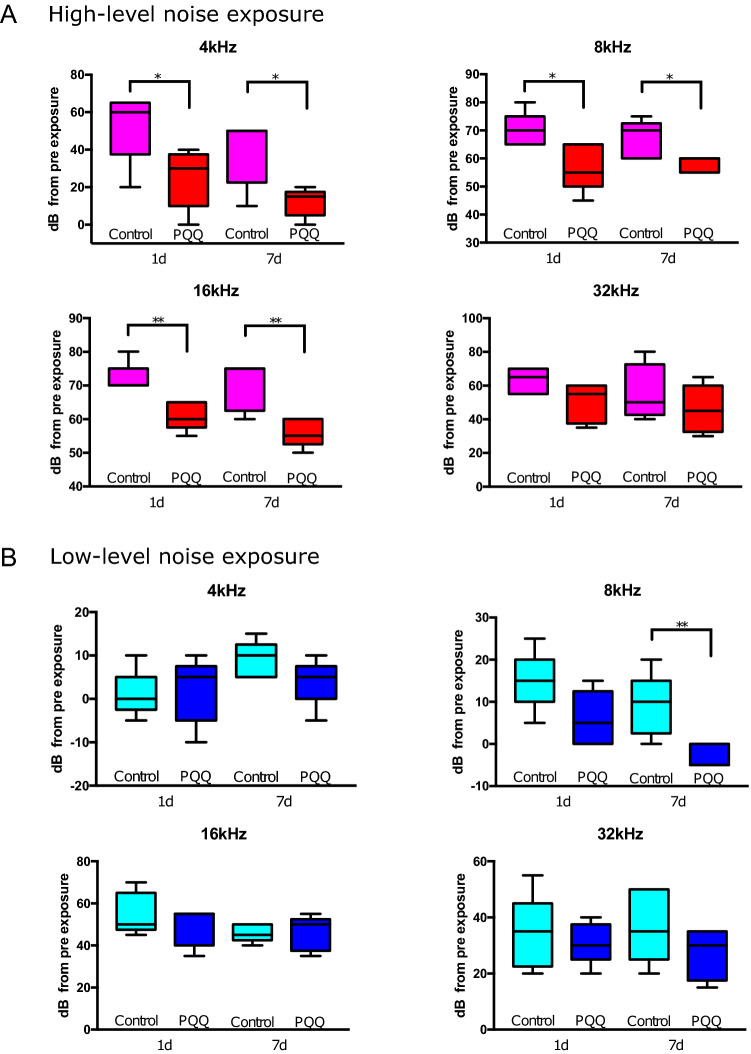
ABR results in the noise-induced hearing loss model (**A**) ABR threshold shifts from pre-exposure thresholds 1 day and 7 days after high-level noise exposure for each frequency (4 kHz, 8 kHz, 16 kHz and 32 kHz). There were significant differences between PQQ-treated animals and the controls at 4 kHz, 8 kHz and 16 kHz. (**B**) ABR thresholds 1 day and 7 days after low-level noise exposure. A significant difference was observed at 8 kHz between low-level noise + PQQ group and low-level noise group. * *p* < 0.05, ** *p* < 0.01. (*n* = 5 per group).

In NIHL experiments, noise exposure was given to 8-week-old C57BL/6 mice with and without PQQ administration. Two sound pressure levels of noise were utilized, based on previous studies^25–27^: 120 dB SPL (high-level noise) or 100 dB SPL (low-level noise) for 4 h. Auditory brainstem response (ABR) thresholds were significantly elevated at all tested frequencies after high-level noise exposure compared to the pre-exposure thresholds (Supplementary Fig. 1A). ABR threshold shifts from pre-exposure thresholds 1 and 7 days after high-level noise exposure were significantly smaller at 4, 8 and 16 kHz in the PQQ-treated group compared to the control group (Fig. 1A).

ABR thresholds were significantly elevated at 16 and 32 kHz after low-level noise exposure compared to the pre-exposure thresholds (Supplementary Fig. 1C). ABR threshold shifts from pre-exposure thresholds 1 day and 7 days after low-level noise exposure tended to be smaller in the PQQ-treated group compared to controls; this difference was statistically significant at 8 kHz 7 days after exposure (*p* < 0.01, Fig. 1B). Vestibular function after noise exposure was also evaluated using vestibular evoked potentials (VsEP). In contrast to the ABR results, VsEP thresholds did not differ significantly between the PQQ-treated group and the control group 7 days after either high-level or low-level noise exposure (*p* > 0.05) (Supplementary Fig. 1B,D). The difference of the amplitude decreases of wave I of the ABR waveform between before and after high-level noise exposure was statistically significant at 4 kHz and the difference of the latency decreases of wave I of the ABR waveform between before and after noise exposure was statistically significant at 4, 8, and 16 kHz after high-level noise exposure and at 8 and 16 kHz after low-level noise exposure, respectively (Supplementary Fig. 2).

**Figure 2:**
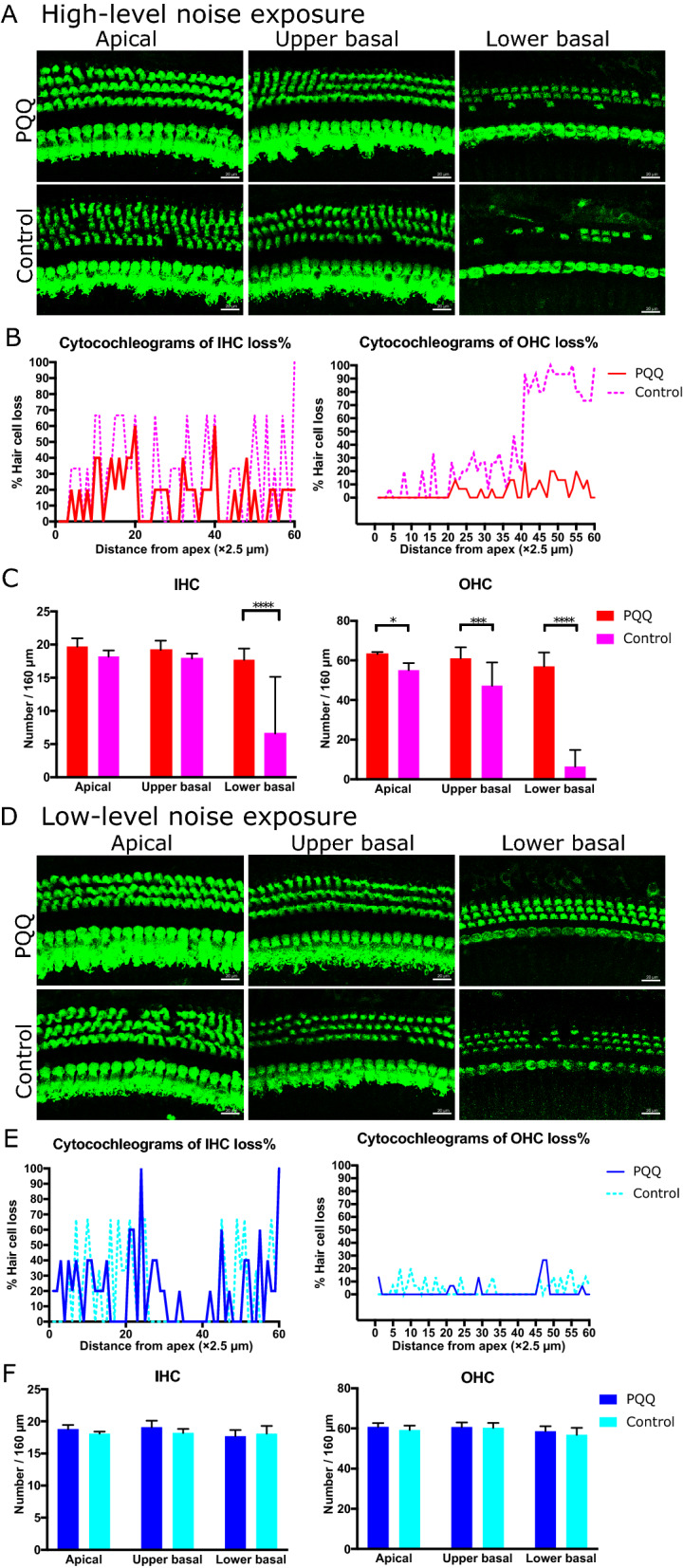
The histological evaluation of hair cells in the noise-induced hearing loss model (**A**) Morphological changes of hair cells after exposure to high-level noise in the apical, upper basal and lower basal turns of the cochlea in the PQQ group and control group. The hair cells are stained with Myo7A (green). Bar, 20 µm. (**B**) The cytocochleograms of IHCs (left) and OHCs (right) for each group. The graphs show the survival rates of IHCs (left) and OHCs (right) as a function of their distance from the apex of the cochlea. (**C**) The numbers of IHCs and OHCs of the sensory epithelium per 160 µm for PQQ and control groups. (**D**)**,** (**E**)**,** (**F**) A, B and C above (respectively) but for low-level noise exposure. * *p* < 0.05, *** *p* < 0.001, **** *p* < 0.0001. (*n* = 5 per group).

Next, we evaluated the histological changes of the cochlea 7 days after high-level or low-level noise exposure by counting the number of inner hair cells (IHCs), outer hair cells (OHCs), ribbon synapses, and SGCs. After high-level noise exposure, the damage to the IHCs and OHCs in the control group was most evident at the lower basal turn of the cochlea, followed by the upper basal turn. Loss of the IHCs and OHCs was also observed, mainly at the lower basal turn, in PQQ-treated animals, but loss of the IHCs and OHCs was significantly attenuated (Fig. 2A and B). The number of the remaining IHCs at the lower basal turn and OHCs at all turns were significantly greater in the PQQ-treated group compared to the controls (Fig. 2C). After low-level noise exposure, loss of the IHCs and OHCs was very limited in both PQQ-treated animals and controls (Fig. 2D and E). There were no significant differences in the number of the remaining IHCs and OHCs between PQQ-treated and control groups (Fig. 2F).

**Figure 3:**
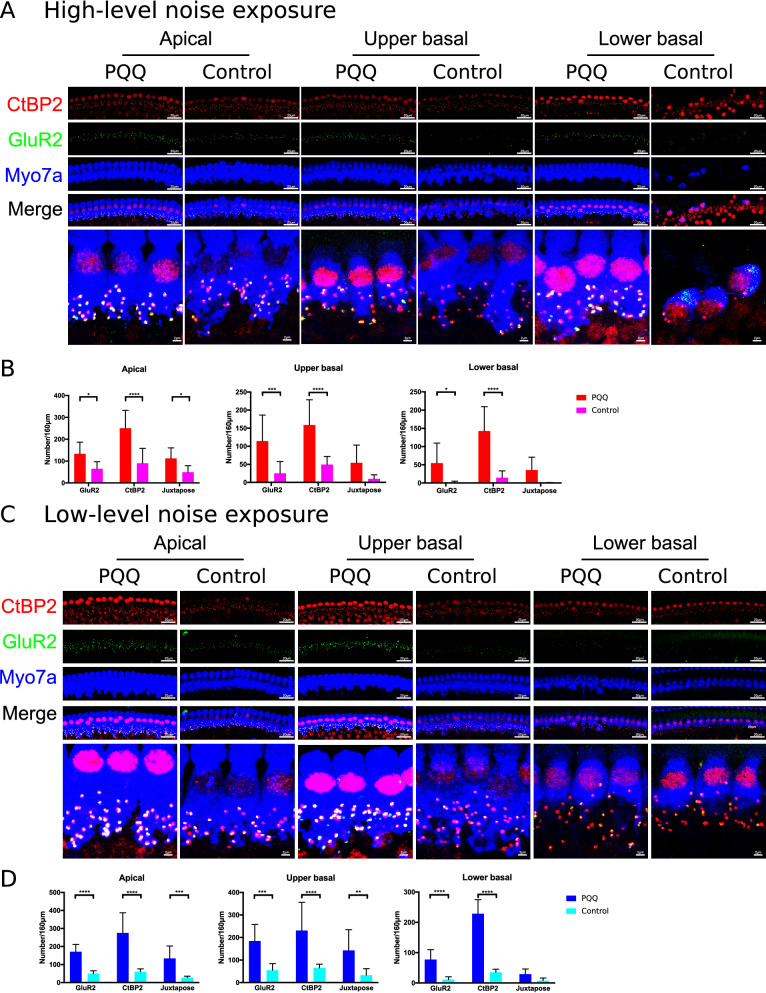
The evaluation of ribbon synapses in the noise-induced hearing loss model **(A)** Confocal images of the ribbon synapses of IHCs of the cochlea in NIHL after exposure to high-level noise. The pre-synaptic marker (CtBP2, red), the post-synaptic marker (GluR2, green) and the hair cell marker (Myo7A, blue) are immunolabeled. Bar, 20 µm, 2 µm in the magnified box. **(B)** The number of ribbon synapses of the IHCs per 160 µm of sensory epithelium. Juxtapose is the number of juxtaposed pre-synaptic marker (CtBP2) and post-synaptic marker (GluR2). **(C,D)** The confocal images of ribbon synapses and the numbers of the ribbon synapses respectively after exposure to low-level noise. * p < 0.05, ** p < 0.01, *** p < 0.001, **** p < 0.0001. (n = 5 per group).

Next, we evaluated the ribbon synapses beneath the IHCs 7 days after noise exposure by immunostaining with a pre-synaptic marker (C-terminal binding protein 2, CtBP2), a post-synaptic marker (Glutamate ionotropic receptor AMPA type subunit 2, GluR2), and a hair cell marker (Myosin 7A, Myo7a) (Fig. 3). After high-level noise exposure, the number of pre-synaptic CtBP2 puncta and postsynaptic GluR2 puncta were both significantly greater at all turns in the PQQ-treated group compared to the control group, and the number of juxtaposed presynaptic ribbons and postsynaptic receptors was significantly greater at the apical turn in the PQQ-treated group compared to the controls (Fig. 3B). Similarly, after low-level noise exposure, the number of pre-synaptic puncta and postsynaptic puncta were both significantly greater at all turns in the PQQ-treated group compared to the controls, and the number of juxtaposed presynaptic ribbons and postsynaptic receptors was significantly greater at the apical and upper basal turns in the PQQ-treated group compared to the controls (Fig. 3D). There was, however, no significant difference in the number of SGCs between PQQ-treated and control groups (*p* > 0.05, Supplementary Fig. 3).

**Figure 4:**
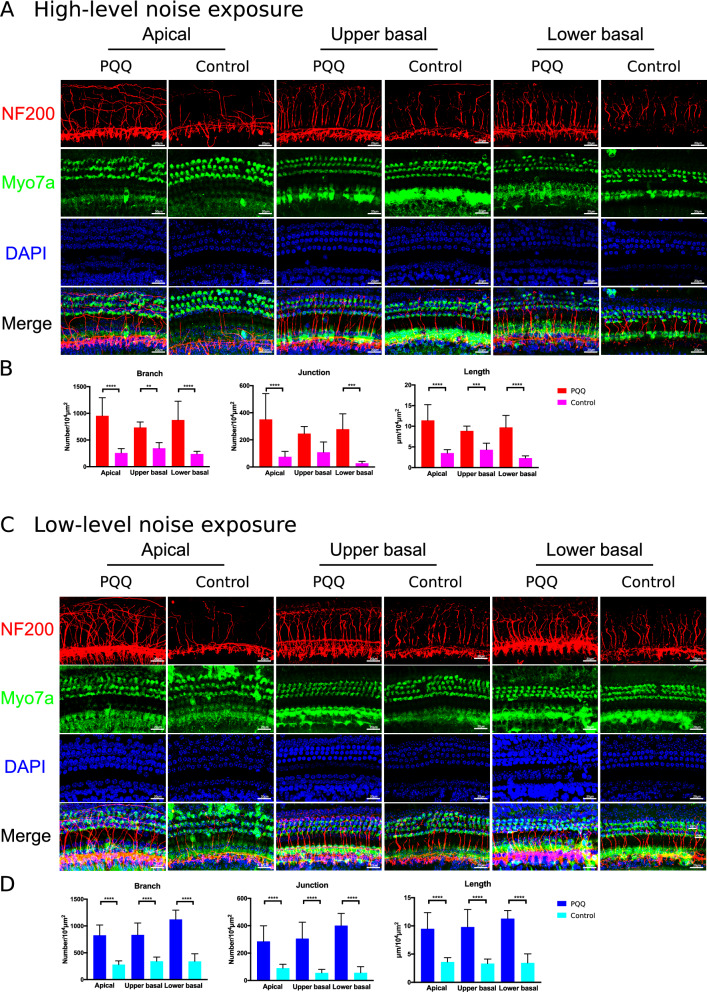
The evaluation of the spiral bundle of the spiral organ of Corti in the noise-induced hearing loss model (**A**) Confocal images of the spiral bundle of the spiral organ at the apical, upper basal and lower basal turns after high-level noise exposure. The hair cells were stained with anti-Myo7A (green), the peripheral axons of the spiral ganglion neurons with anti-NF200 (red), and the nuclei with DAPI (blue). Bar, 20 µm. (**B**) Quantitative analysis result of SGC peripheral axons with branches, junctions and lengths after high level noise exposure. The description of each parameter is in the methods section. (**C, D**) The spiral bundle of the spiral organ and the quantitative analysis of the bundles respectively after low-level noise exposure. ** *p* < 0.01, *** *p* < 0.001, **** *p* < 0.0001. (*n* = 5 per group).

Next, we evaluated the cochlear nerve fibers beneath the HCs 7 days after noise exposure by immunostaining with NF200 and analyzing the skeletonized image (Fig. 4). High-level noise exposure induced severe degeneration of the spiral bundles in the controls, but such degeneration was attenuated by PQQ treatment; the numbers of the branches and junctions and the length of the spiral bundles were significantly greater at all turns in the PQQ-treated group compared to the controls, except for the number of junctions at the upper basal turn after high-level noise exposure (Fig. 4B). Similarly, the significant protective effect of PQQ on the spiral bundles was also observed after low-level noise exposure (Fig. 4D).

Collectively, all the physiological and histological results indicate that PQQ has a protective effect against noise-induced cochlear damage.

### PQQ protected against age-related hearing loss

**Figure 5:**
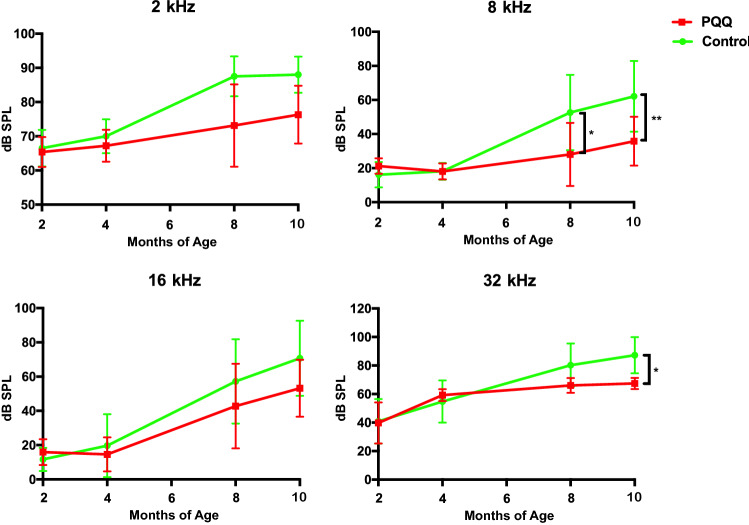
ABR thresholds in the age-related hearing loss model ABR thresholds of each frequency (4 kHz, 8 kHz, 16 kHz and 32 kHz) at each month of age. There were significant differences between the PQQ and control groups at 8 kHz and 32 kHz at 10 months of age. * *p* < 0.05, ** *p* < 0.01. (Control group (*n* = 10) and PQQ group (*n* = 11)).

The age-related deterioration of hearing function was evaluated using the ABR (Fig. 5). ABR thresholds gradually increased with age at all tested frequencies in both controls and PQQ-treated animals, but the extent of the increase was smaller in the PQQ-treated animals. At 10 months of age, ABR thresholds were lower at all frequencies in the PQQ-treated group compared to the controls, with the difference being statistically significant at 8 kHz and 32 kHz, indicating that PQQ suppressed hearing decline associated with aging. VsEP thresholds were not significantly different, however, between PQQ-treated and control groups at 10 months of age (Supplementary Fig. 4).

**Figure 6:**
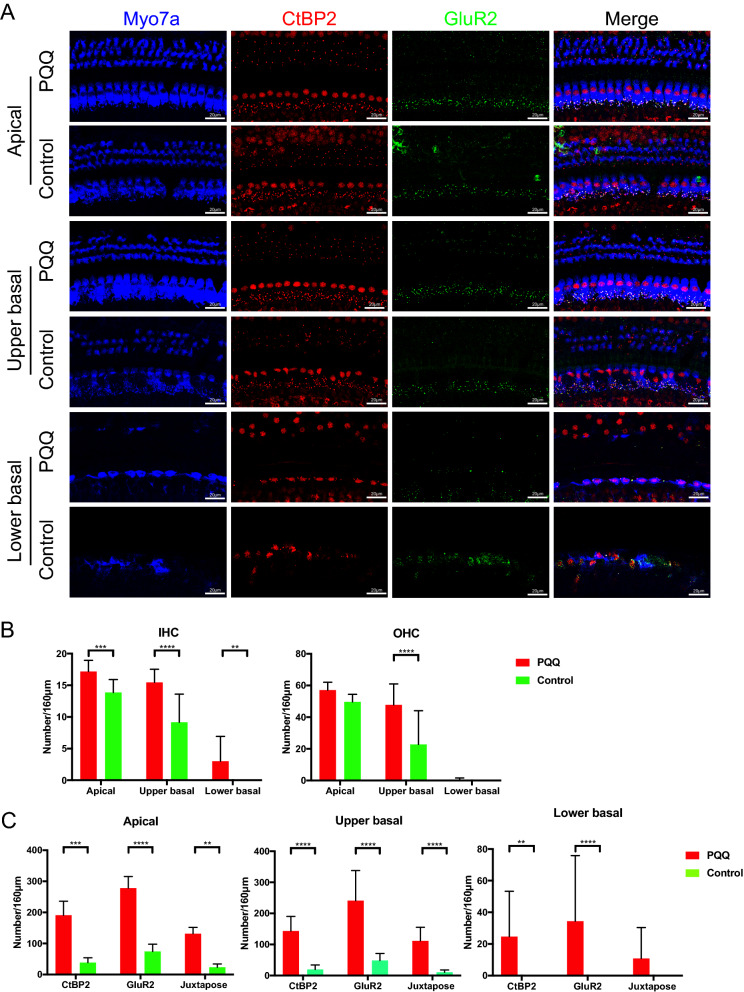
Evaluation of the hair cells and ribbon synapses in the age-related hearing loss model (**A**) Confocal images of the ribbon synapses of the IHCs of the cochlea in ARHL. The pre-synaptic marker (CtBP2, red), the post-synaptic marker (GluR2, green) and the hair cell marker (Myo7A, blue) are immunolabeled. (**B**) The number of IHCs and OHCs of the sensory epithelium per 160 µm. (**C**) The number of the ribbon synapses of the IHCs per 160 µm of sensory epithelium. Juxtapose is the number of juxtaposed pre-synaptic marker (CtBP2) and post-synaptic marker (GluR2). ** *p* < 0.01, *** *p* < 0.001, **** *p* < 0.0001. (Control group (*n* = 10) and PQQ group (*n* = 11)).

Next, we evaluated histological changes in the cochlea at 10 months of age using the surface preparation method with immunostaining (Fig. 6). In the control animals, the IHCs had virtually disappeared at the lower basal turn and were markedly damaged at the apical and upper basal turns (Fig. 6A). This loss of IHC was significantly attenuated at all turns by PQQ treatment (Fig. 6B). The OHCs were also significantly damaged in the control animals, with the lower basal turns being the most severely affected. PQQ treatment attenuated loss of OHC at the apical and upper basal turns, with the difference being statistically significant at the upper basal turn (*p* < 0.0001) (Fig. 6B). The numbers of CtBP2 and GluR2 puncta, as well as the number of juxtaposed presynaptic ribbons and postsynaptic receptors, were reduced markedly at all turns of the cochlea in the controls. PQQ-treatment significantly attenuated damage to the pre-synaptic CtBP2 puncta and post-synaptic GluR2 puncta at all turns, except the juxtaposed presynaptic ribbons and postsynaptic receptors at the lower basal turn (Fig. 6C).

**Figure 7:**
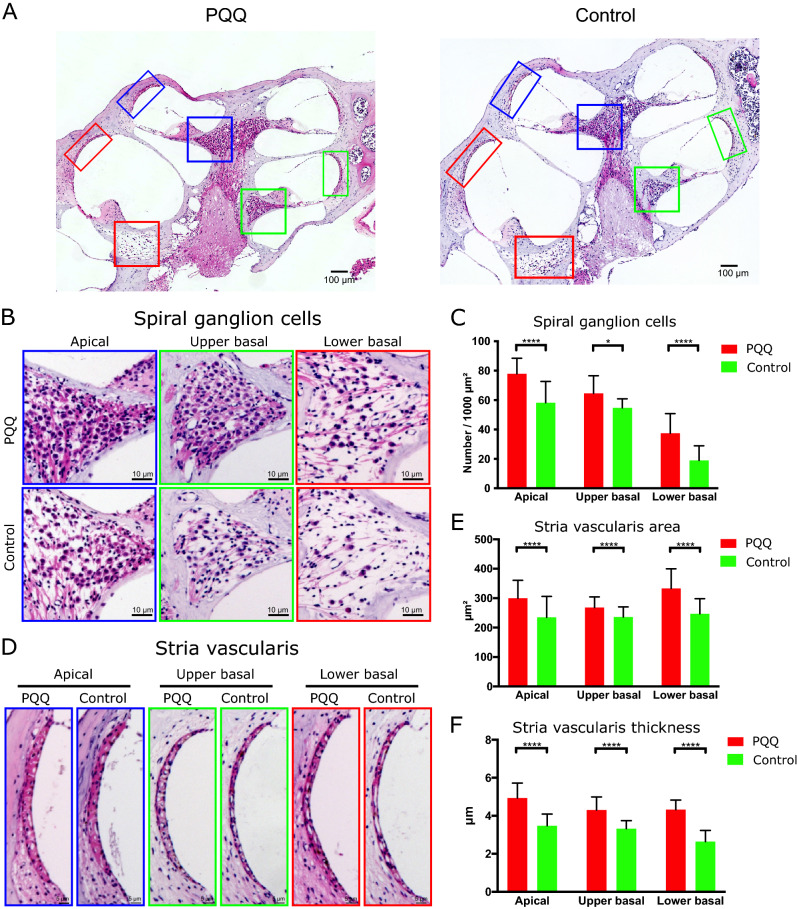
Histological evaluation of the spiral ganglion and the stria vascularis in the age-related hearing loss model (**A**) The histological H-E images of the cochlea in each group showing the spiral ganglion cells (SGCs) (square) and the stria vascularis (SV) (rectangle) at apical (blue), upper basal (green) and lower basal (red) turns. Bar, 100 µm. (**B**) SGCs. Bar, 10 µm. (**C**) The densities of SGCs per 1000 µm^2^. (**D**) The SV. Bar, 5 µm. (**E, F**) The area (**E**) and the thickness (**F**) of the SV. * *p* < 0.05, **** *p* < 0.0001. (Control group (n = 10) and PQQ group (*n* = 11)).

We also evaluated the number of SGCs and the area and thickness of the SV at 10 months of age using hematoxylin eosin (H-E) staining of serial sectioning (Fig. 7). The number of SGCs was reduced at all turns in the controls, most severely at the lower basal turn (Fig. 7C). This tendency that the SGCs were more severely damaged at the lower turn was also observed in PQQ-treated animals, but the reduction of the SGCs due to aging was significantly attenuated at all turns by PQQ treatment. The area and thickness of the SV were also significantly larger at all turns in the PQQ-treated group compared to controls (*p* < 0.0001) (Fig. 7E,F).

Collectively, all these results indicate that PQQ protects cochlear tissues from the degeneration due to aging and attenuates ARHL.

## Discussion

The current study demonstrated the protective effects of PQQ on the cochlea, both physiologically and histologically, from noise-induced and age-related damage. PQQ significantly reduced the damage to a variety of cochlear tissues, such as the IHCs, OHCs, synapse ribbons, nerve fibers, SV, and the SGCs. One of key factors of auditory protective compounds is their multifaceted protective effect because several factors are involved in hearing loss including degeneration of the HCs, SGCs, SV and blood vessels^14^. Therefore, PQQ may be a promising compound to be used clinically for preventing NIHL and ARHL.

The mechanisms underlying NIHL and ARHL include mitochondrial free radical formation^8,28,29^, which actively stimulates intracellular stress pathways, leading to programmed and necrotic cell death^9^. The mechanisms by which the protective effects of PQQ are conferred are considered to be mainly based on the ROS scavenging function^30,31^, but there may be other putative mechanisms. It has been shown that PQQ promotes mitochondrial biogenesis^32^. PQQ controlled the redox processes in the mitochondrial respirator chain and attenuated oxidative stress in mitochondria^33^. The supplementation of PQQ in the diets of mice and rats improved the mitochondrial contents and lipid metabolism^22^, and improved the respiratory quotient by increasing the amount of mitochondria and their function^23^. In humans, supplementation with PQQ recovered the antioxidant potential, attenuated inflammatory responses, and increased urinary metabolites related to mitochondrial functions^21^. In in vivo models, PQQ prevented the rotenone-induced neurotoxicity in Parkinson’s disease models in rats by promoting mitochondrial function and regulating mitochondrial fission and fusion^24^ via the activation of adenosine monophosphate-activated protein kinase (AMPK) signaling pathway^34^. It also ameliorated spinal cord injury by attenuating the gene expression of inducible nitric oxide synthase in rat^35^, protected the brain from reversible middle cerebral artery occlusion in rat^36^ and promoted the regeneration of rat sciatic nerve^37^. It is known that the SV is most commonly affected by mitochondrial dysfunction, followed by the HCs and SGCs^38^. In addition, the mitochondrial function in House Ear Institute-Organ of Corti 1 (HEI-OC1) auditory cell lines was protected by PQQ pretreatment in an H_2_O_2_-induced senescence model and is associated with the SIRT1/PGC-1α signaling pathway^39^. The protective effect of PQQ in mitochondrial biogenesis is also expected in inner ear tissue, suggesting that PQQ can also work protectively in the auditory organs.

In the current study, there was no significant difference in the number of SGCs under NIHL between PQQ-treated animals and controls. Since the degeneration of SGCs after noise exposure occurs over several weeks, the observation at 7 days after noise exposure employed in the current study may not be sufficient to observe significantly reduced numbers of SGCs. In a study evaluating the interaction between NIHL and ARHL, in which CBA/CaJ mice were exposed to 8–16 kHz octave band 100 dB SPL noise for 2 h, a significant decrease in the number of SGCs was observed in mice exposed to noise at 6 weeks of age and assessed at 96 weeks of age, but the number of SGCs was not significantly decreased when assessed 2 weeks after noise exposure either at 6 or 96 weeks of age^40^. In addition, the evaluation of SGC loss from the aspect of cochlear synaptopathy, called hidden hearing loss, under NIHL and ARHL was studied by the same group and SGC loss was found to be highly dependent on synaptic damage^26^. In animals exposed to the same noise as above for 2 h, the number of SGCs had decreased by 25–40% at 88 weeks post-exposure, but only by 5–10% at 32 weeks post-exposure. In animals exposed to slightly weaker noise (8–16 kHz octave band 91 dB SPL) for 2 h, there was no decrease in the number of SGCs at 32- and 48-weeks post-exposure. In another study, a significant decrease in the number of SGCs was observed 2 weeks after noise exposure in both TTS (108 dB SPL 1–8 kHz broadband noise for 5 min) and PTS models (116 dB SPL 2–8 kHz broadband noise for 2 h) in C57BL/6 mice at 6 weeks of age^41^.

The protective effect of PQQ to hair cell loss was observed in high-level noise exposure, whereas the protective effect was limited to the ribbon synapses in low-level noise exposure. The low-level noise exposure was designed primarily to assess NIHL due to synaptopathy and was not expected to be enough to cause hair cell loss.

The hair cell loss in high-level noise exposure was most evident at the lower basal turn of the cochlea, followed by the upper basal turn. It has been reported that ROS spread from the basal to the apex after noise exposure causing necrosis or apoptosis^7,42–44^ and that the HCs in the basal turn is more susceptible to ROS compared to more apical turns and can be protected by the ROS scavengers^45^. The regional difference observed in the current study was consistent with these previous studies.

It is important to determine the optimum daily dose of PQQ for clinical purposes. While most studies evaluating the effects of PQQ have shown protective effects, a high concentration of PQQ has been reported to cause cell toxicity in cell culture evaluation^46^. In the current study, for the NIHL group we adopted a concentration of PQQ at 4 mg/kg/day for 2 weeks and injected an additional 20 mg/kg subcutaneously and for the ARHL group we used a concentration of PQQ at 4 mg/kg/day for 8 months. At these dosages, we did not observe body weight decrease due to PQQ. It has been reported that sub-chronic toxicity was not induced when PQQ was administered via the oral route at doses of up to 400 mg/kg/day^47^, whereas acute toxicity was induced when doses of 1000–2000 mg/kg/day were administered in male rats or 500–1000 mg/kg/day in female rats^48^. In a study examining whether oral supplementation of PQQ at 5 or 20 mg/kg/day for 14 days repaired glucose tolerance in type 2 diabetic KK-A^y^ mice^49^, the 20 mg/kg/day group did not show drastically better improvement compared to the 5 mg/kg/day group. Therefore, we did not increase the dose above 5 mg/kg/day. Regarding these previous studies, many levels of dosage have been used for the in vivo evaluation of PQQ. PQQ was effective for preventing neurodegeneration caused by oxidative stress when 20 mg/kg/day of PQQ was given to rats^50^. The early supplementation of PQQ at 0.3 or 0.96 mg/kg/day had persistent long-term protective effects on the developmental programming of hepatic lipotoxicity and inflammation in obese mice^51^. Both cognitive function and memory function were improved with the oral route administration of PQQ at 20 mg/kg for 2 or 4 weeks in aging rats^52^. An intraperitoneal injection of PQQ at 5 mg/kg/day for 1 week protected against secondary damage following primary physical injury to the spinal cord^35^. PQQ at 20 mg/day (about 0.3 mg/kg/day) for 12 weeks prevented reduction of brain function in aged persons, especially in attention and working memory^53^. Considering these varieties of doses used in ours and previous studies, PQQ may have a relatively wide dose range to achieve optimal efficacy.

The vestibular function evaluated with VsEP at 7 days after noise exposure showed no significant difference between the PQQ-treated group and the control group. The vestibular dysfunction after noise exposure was observed in previous studies withVsEP evaluation^54^, in which noise intensity ranged from 110 dB SPL^55^ or 120 dB SPL^56–58^ to 158 dB SPL^59^ or 160 dB SPL^60^. The condition of noise used in the current study was within this range. Although we did not evaluate the change of VsEP itself due to noise exposure in the current study, the protective effect of PQQ to noise-induced vestibular damage is considered to be small.

The vestibular function evaluated with VsEP at 10 months of age in ARHL model also showed no significant difference between the PQQ-treated group and the control group. The age-dependent vestibular dysfunction evaluated with VsEP and histological analysis has been reported in CBA/CaJ mice^61^ , A/J mice^62^, and C57BL/6 mice^59^, in which the changes in VsEP threshold were small (0.220 dB/month^62^ or 0.11 dB/month^59^). The expected threshold increase within 10 months in the current study was relatively small compared to the measurement threshold interval (5 dB). Therefore, the evaluation in the longer timespan may be required to confirm the protective function of PQQ to vestibular organs, even if it is present.

One limitation of this study is that the PQQ distribution in the cochlea was not analyzed. The drug distribution is an important factor in determining the optimum concentration. If the compounds metabolized rapidly and the concentration in the target tissue is not high enough, the administration should include a sustained release formulation. It has been reported that PQQ is readily absorbed in the lower intestine with 81% of the absorbed dose then excreted by the kidneys within 24 h of oral administration. Six hours after administration, PQQ was found to be distributed in urine (62.1%), liver (5.4%), red blood cells (10.6%), and skin (0.3%) with most retention occurring in the kidneys and skin at 24 h after administration^63^.

Another limitation of this study is that C57BL/6 mice was adopted in the evaluation of ARHL. Although C57BL/6 mice show ARHL phenotype, the mutation in Cadherin 23 (Cdh23) gene partly contributes to ARHL^64,65^, whose mechanisms differ from common pathways in aging including oxidative stress formation. C57BL/6 mice is utilized in some studies regarding ARHL, whereas the evaluation in multiple strains is needed to clarify the effect and the molecular pathways of PQQ in ARHL.

## Conclusion

The physiological and histological results presented demonstrate that PQQ has a protective effect on the auditory system from NIHL and ARHL in mice. Considering that PQQ can be given safely to humans, it can be a useful therapeutic drug for the treatment and prevention of NIHL and ARHL in humans.

## Materials and methods

### Experimental animals

Eight-week-old male C57BL/6 mice (weighing 28–30 g) were used. Normal Preyer’s reflex was confirmed in all mice before experimentation. Mice were housed in a light–dark cycle (12:12) with free access to water and food. All procedures regarding the use and care of animals were approved by the Institute for Animal Care and Use Committee of Medical Science, University of Tokyo (I-P19-058). All methods were performed in accordance with the relevant guidelines and regulations including the ARRIVE guidelines (Animal Research: Reporting of In Vivo Experiments).

### Experimental protocol

The timelines of the experiments are shown in Supplementary Fig. 5. The specific protocols for drug administration, noise exposure and measurements are described as follows.

### Drug administration

PQQ disodium salt originated from the Mitsubishi Gas Chemical Company, Inc. (Tokyo, Japan). The initial dose of PQQ at 2 mg/kg/day was chosen based on a previous study evaluating mitochondrial function, lipid and energy metabolism in rats^22^, however the concentration was doubled in case the freely-drinking (not via oral gavage) mice did not drink the PQQ-containing water because of the taste or other reasons. The concentration of PQQ drinking water was 2.4 mg/100 mL and the 4 mg/kg/day dose value was converted using the following values based on our basic data: the body weight was 30 g/mouse, and the amount of drinking water was 5 mL/day/mouse. These basic values are similar to other studies: the amount of drinking water was 6 mL/day/mouse for C57BL/6 J mouse^66^. The actual values of feeding water consumed were individually determined for each cage (Supplementary Fig. 6 and 7).

In the NIHL experiment, PQQ was prepared at 2.4 mg/100 mL for drinking in water. Mice were divided into control groups (*n* = 5 for high-level noise and *n* = 5 for low-level noise) and PQQ groups (*n* = 5 for high-level noise and *n* = 5 for low-level noise). Mice in the control groups were fed with normal drinking water, and normal saline was injected subcutaneously 1 h before the noise exposure. Mice in the PQQ group were fed with drinking water containing PQQ from 7 days before the noise exposure to 7 days after the noise exposure, and 0.01 mL/g (BW) PQQ in normal saline (2 mg/mL) was injected subcutaneously once (20 mg/kg) at 1 h before noise exposure.

In the ARHL experiment, mice were divided into a control group (*n* = 10) and a PQQ group (*n* = 11), the latter of which were fed with drinking water containing PQQ at 2.4 mg/100 mL. There was no significant difference in body weight between the PQQ-treated and control groups at any age (Supplementary Fig. 8).

### Noise exposure

The protocol of noise exposure was based on our previous report^25^. The high-level noise exposure was designed primarily to assess NIHL due to ROS-dependent cytotoxicity, while the low-level noise exposure was designed primarily to assess NIHL due to synaptopathy rather than hair cell loss^67^. Animals were exposed to noise in an illuminated and ventilated sound exposure chamber. The sound exposure chamber was equipped with a loud horn speaker (PSD:3006; Eminence Speaker LLC, Eminence, United States), which was driven by a sound player (DN-F300; DENON, Kawasaki, Japan) and a power amplifier (P7000S; YAMAHA, Hamamatsu, Japan). Sound levels were measured at multiple locations within the sound chamber using a calibrated condenser microphone (UC-31; RION, Tokyo, Japan) and sound level meter (NA-42; RION, Tokyo, Japan) to ensure the uniformity of the stimulus. The stimulus intensity varied by a maximum of 2 dB across measured sites within the exposure chamber. The sound levels were also measured during the exposure to ensure stability. Animals in stainless-steel wire cages (10 × 10 × 10 cm) were individually subjected to 8 kHz octave band noise at 120 dB SPL (high-level noise) for 4 h or 100 dB SPL (low-level noise) for 4 h. All animals were exposed at 09:00 am to reduce any circadian rhythm variations, which may influence the threshold shifts.

### Auditory brainstem response measurement

The protocol of auditory brainstem response (ABR) measurement was based on our previous reports^25,68^. Mice were anesthetized with a mixture of xylazine hydrochloride (10 mg/kg) and ketamine hydrochloride (40 mg/kg). Needle electrodes were inserted subcutaneously at the vertex (active electrode), beneath the pinna of the measured ear (reference electrode), and beneath the opposite ear (ground electrode). The sound frequencies delivered were 4, 8, 16 and 32 (actual value: 31.25) kHz in the NIHL experiment and 2, 8, 16 and 32 (actual value: 31.25) kHz in the ARHL experiment and the stimulus repetition rate was 20 Hz, each with a duration of 5 ms, which consisted of a rise/fall time of 1 ms and a 3 ms plateau. The 500 responses were bandpass filtered (0.4–32 kHz) and averaged with the Neuropack MEB-2208 measuring system (Nihon Kohden, Tokyo, Japan). The threshold was defined as the lowest intensity level at which a clear reproducible waveform was visible in the trace. The sound intensity was varied in 5 dB intervals near threshold. In the NIHL experiment, the ABR was recorded at 7 days before noise exposure, and at 1 and 7 days after noise exposure. In the ARHL experiment, ABR was recorded at 2, 4, 8 and 10 months of age.

### Vestibular evoked potential measurement

The protocol of vestibular evoked potential (VsEP) measurement was based on our previous report^68^. The VsEP is a compound action potential of the vestibular nerve and central relays that is elicited by linear acceleration ramps applied to the cranium^69^, and can evaluate vestibular function in mice. The VsEP measurement system consisted of an acceleration stimulation system and the evoked potential measuring system. Under general anesthesia with a mixture of xylazine hydrochloride (10 mg/kg) and ketamine hydrochloride (40 mg/kg), the head of the mouse was fixed on a stimulation frame system using a SG-4 N mice head holder (Narishige, Tokyo, Japan). Two types of vibration acceleration, symmetric parabolic waves with ramps (SPR) and symmetric parabolic waves with linear acceleration and ramps (SPLR) (Supplementary Information), were used to stimulate the head holder. The acceleration stimulation was generated with a WaveMaker Mobile S-0105 vibration exciter (Asahi Seisakusho, Tokyo, Japan) by an in-house designed direct current power amplifier. The acceleration was measured with a 352C65 accelerometer and 482A21 signal conditioner (PCB Piezotronics, Depew, United States) which was used to calibrate the peak jerk (expressed in 0 dB re. 1 g/ms) of the VsEP stimulation. The evoked potential measurement system was identical to the ABR measurement system, and the typical waveforms, with a latency of 1 to 2 ms (peak jerk shape), were regarded as effective responses^69^. The intensity was varied in 5 dB intervals near threshold. In the NIHL experiment, VsEP was recorded at 7 days after noise exposure. In the ARHL experiment, VsEP was recorded at 10 months of age.

### Immunohistochemistry preparation

The protocol of immunohistochemistry preparation was based on the previous reports^70,71^. Mice were decapitated after the final measurement of ABR and VsEP, and the inner ears were removed and fixed by immersion in 4% formaldehyde overnight at 4 °C. The inner ears were decalcified in 10% EDTA for 1 week, and processed with cochlear surface preparation^72,73^. After washing three times with phosphate-buffered saline (PBS), the tissues were blocked with 0.3% Triton X-100 and 5% bovine serum albumin (BSA) for 1 h, stained with Myo7A (25–6790, Proteus) (1:200) and NF200 (N0142, Sigma-Aldrich) (1:200) or Myo7A, GluR2 (MAB397, Chemicon) (1:1000) and CtBP2 (612044, BD Biosciences) (1:200) in 0.3% Triton X-100 and 5% BSA overnight at 4 °C. The next day, after washing three times with PBS, the tissues were stained with anti-mouse IgG (Alexa Flour 568 A11004, Thermo Fisher Scientific) (1:200) and anti-rabbit IgG (Alexa Fluor 488 A11008, Thermo Fisher Scientific) (1:200) or anti-rabbit IgG (Alexa Fluor 405 A31556, Thermo Fisher Scientific) (1:200), anti-mouse IgG2a (Alexa Fluor 488 ab172324, Abcam) (1:1000) and anti-mouse IgG1 (Alexa Fluor 568 A21124, ThermoFisher) (1:1000) in 0.3% Triton X-100 and 5% BSA for 1 h at room temperature. VECTASHIELD Mounting Medium with or without DAPI (H-1200, H-1000, Vector Laboratories) was used for the slide embedding after washing three times with PBS. The fluorescent images were collected using a confocal microscope system (A1R, Nikon, Japan) with a 60 × (NA 1.4) oil immersion lens.

### Image analysis of ribbon synapses and the nerve fiber network

Processing and analysis of immunohistochemistry images were performed using NIS-Elements (Nikon, Japan), ImageJ with Fiji, Skeletonize3D and Analyze Skeleton plugins^74–76^. The number of ribbon synapses immunostained for CtBP2, GluR2 and juxtaposed was counted using the Analyze Particles Plugin. The images of nerve fibers were skeletonized using standard processing operations in ImageJ/FIJI (medial axis transform), which involved an intensity threshold, followed by thinning and then pruning of the objects. The skeleton was vectorized to identify and count/measure branches (skeletal backbone), end points and branch points as graphic vectors/points. The following parameters represent the features of network nodes: branch count (the number of branches attached to branch points), junctions (the number of points where three or more branches are attached), average branch length (the average length of all the branches in one image).

### Histological preparation

The preparation and examination protocols for determining cochlear pathology were based on our previous reports^25,68^. Mice were decapitated, and the inner ears were removed and fixed by immersion in 4% formaldehyde overnight at room temperature. The inner ears were decalcified in 10% EDTA for 1 week, dehydrated and embedded in paraffin for cutting with the following steps: 100% ethanol, 7 changes, 1.5 h each, 100% xylene, 3 changes, 45 min each, and paraffin wax (56–58 °C), 2 changes, 1.5 h and 2 h. Tissue slices were collected on SuperFrost slides (Matsunami Glass Ind.,Ltd., Osaka, Japan), dried on a heating plate at 37 °C overnight and stained with hematoxylin eosin (H-E). The images were captured with an all-in-one fluorescence microscope (BZ-X710, KEYENCE, Osaka, Japan).

### Image analysis of spiral ganglion cells

The number of the spiral ganglion neurons and the thickness and the area of the SV were evaluated using H-E stained serial sections. The observer-blinded counting of the spiral ganglion neurons at each of the apical, upper basal and lower basal turns of the cochlear was performed on mid-modiolar sections obtained from seven non-overlapping 28 μm sections in each cochlea. The thickness and the area of the SV were calculated using ImageJ with Fiji plugins. The H-E stained images were processed with the Colour Deconvolution plugin, then the regions of the SV were identified using the Auto Threshold plugin in the hematoxylin-stained image. The area and the thickness of the SV was then calculated using the Analyze plugin.

### Statistical analysis

All measurements were completed by the same examiner but the findings were analyzed in a blinded manner. Excel for Microsoft 365 (https://www.microsoft.com/microsoft-365/excel, version 2207, Microsoft, Redmond, WA, USA) was used for processing data. ABR thresholds, VsEP thresholds and HC counting results were analyzed with one-way analysis of variance (ANOVA) followed by Bonferroni’s multiple comparison testing using Prism (https://www.graphpad.com/scientific-software/prism/, version 7.0 for Apple Macintosh, GraphPad Software, San Deigo, CA, USA). Data were presented as mean ± standard deviation. A confidence level of 95% was considered statistically significant. * *p* < 0.05, ** *p* < 0.01, *** *p* < 0.001, **** *p* < 0.0001 were used to depict the significance level in bar graphs.

## Supplementary Information

Supplementary Information.

## Data Availability

The datasets used and analyzed during the current study are available from the corresponding author on reasonable request.
