# Supplementary material for: Effects of pyrroloquinoline quinone on noise-induced and age-related hearing loss in mice
Source: Sci Rep. 2022 Sep 23;12:15911. doi: 10.1038/s41598-022-19842-w (PMC9508078; doi:10.1038/s41598-022-19842-w)
Supplement: Supplementary file 1 — Supplementary Information. [file 41598_2022_19842_MOESM1_ESM.docx]

**Supplementary Information**

**Supplementary Figure 1**

ABR and VsEP results in the noise-induced hearing loss model.

A: ABR thresholds for each frequency (4 kHz, 8 kHz, 16 kHz and 32 kHz) before and 1 and 7 days after high-level noise exposure in PQQ-treated animals and the controls. ABR thresholds were significantly elevated at all tested frequencies after high-level noise exposure compared to the pre-exposure thresholds in both groups (p<0.05; * marks are not shown).

B: VsEP thresholds measured 7 days after high-level noise exposure in PQQ-treated animals and the controls.

C: ABR thresholds for each frequency (4 kHz, 8 kHz, 16 kHz and 32 kHz) before and 1 and 7 days after low-level noise exposure in PQQ-treated animals and the controls. ABR thresholds were significantly elevated at 16 and 32 kHz after low-level noise exposure compared to the pre-exposure thresholds in both groups (p<0.05; * marks are not shown).

D: VsEP thresholds measured 7 days after low-level noise exposure in PQQ-treated animals and the controls.

HN: high-level noise (120 dB SPL for 4 hours), LN: low-level noise (100 dB SPL for 4 hours), SPR: Symmetric parabolic waves with ramps, SPLR: Symmetric parabolic waves with linear acceleration and ramps.

* p<0.05, ** p<0.01. (n=5 per group)

**Supplementary Figure 2**

The amplitude decrease and the latency decrease of wave I of the ABR waveform between before and after high-level (HN) or low-level noise (LN) exposure.

* p<0.05, ** p<0.01, **** p<0.0001. (n=5 per group)

**Supplementary Figure 3**

Histological evaluation of the spiral ganglion cells in the noise-induced hearing loss model.

H-E stained images show the spiral ganglion neurons (SGN) after high-level (HN) or low-level noise (LN) exposures in PQQ-treated animals and the controls. Bar, 10µm. The graphs show the densities of SGN per 1000 µm².

ns: not significant (p>0.05) (n=5 per group)

**Supplementary Figure 4**

VsEP thresholds measured at 10 months of age in PQQ-treated animals and the controls.

(control group (n=10) and PQQ group (n=11))

**Supplementary Figure 5**

The timeline of the experiments. A: Noise-induced hearing loss model. B: Age-related hearing loss model.

**Supplementary Figure 6**

The conversion of PQQ consumption per animal per day, calculated from the amount of water consumed in each cage, is shown. Drinking water was changed every three or four days and the amount consumed was measured at each change. The x axis indicates the total number of water changes since the first change. Although there was some variation from week to week, the consumption was around 0.1 mg per animal per day.

**Supplementary Figure 7**

The converted average of the feeding amount for all cages is shown. The x-axis represents the total number of times the cage was changed, which was every 3 or 4 days, after the first change. The amount of PQQ consumed was assessed at each cage change. The daily PQQ intake of mice was approximately 4 mg/kg per animal. (n=5)

**Supplementary Figure 8**

Mean body weight (g) of mice from 2 to 10 months of age in the ARHL experiment, showing no significant difference between the control and PQQ-treated group at any age.

(control group (n=10) and PQQ group (n=11))

**Supplementary Data 1**

**VsEP acceleration waveform**

The functions for the VsEP acceleration were calculated as follows:

The symmetric parabolic waves with ramps (SPR):

For t = 0 ms to 20 ms: f(t) = −x/20

For t = 20 ms to 21 ms: f(t) = (x − 20) ^2− 1

For t = 21 ms to 22 ms: f(f) = −(x − 22) ^2 + 1

For t = 22 ms to 42 ms: f(t) = −(x − 42)/20

The trigger for the evoked potentials was at t = 20.

The symmetric parabolic waves with linear acceleration and ramps (SPLR):

For t = 0 ms to 20 ms: f(t) = −x/20

For t = 20 ms to 20.5 ms: f(t) = (x − 20) ^2− 1

For t = 20.5 ms to 22 ms: f(f) = (x − 21) − 0.25

For t = 22 ms to 22.5 ms: f(t) = −(x − 22.5) ^2 + 1

For t = 22.5 ms to 42.5 ms: f(t) = −(x − 42.5)/20

The trigger for the evoked potentials was at t = 20.

These waveforms provide 1 ms rise/fall triangle wave jerk acceleration, or 0.5 ms rise/fall - 1.5 ms flat jerk stimulation in theory, however the actual output depends on the resonant frequencies of the stimulation system. The values of f(t) are converted to the full scale of the D/A converter. All waveforms are produced with the WF1947 function generator and ported to the Arduino based function generator with external triggers. The codes and associated data are available at https://sourceforge.net/projects/ardufgx/.
